# Characterization of the volatile organic compounds produced from green coffee in different years by gas chromatography ion mobility spectrometry

**DOI:** 10.1039/d2ra01843h

**Published:** 2022-05-23

**Authors:** Chen Min, Mai Biyi, Lu Jianneng, Li Yimin, Liu Yijun, Cheng Long

**Affiliations:** Hainan Key Laboratory of Storage & Processing of Fruits and Vegetables, Agricultural Products Processing Research Institute, Chinese Academy of Tropical Agricultural Sciences No. 48 Renmindadaonan Zhanjiang 524001 China liuyijun-1@163.com +86 759 2208758 +86 759 2221090; Key Laboratory of Tropical Crop Products Processing of Ministry of Agriculture and Rural Affairs Zhanjiang 524001 China; College of Tropical Crops Institute, Yunnan Agricultural University Kunming 650201 China; Modern Agricultural Development Co., Ltd of Zhanjiang Agribusiness Group No.35 Renmin Avenue Middle Zhanjiang 524258 China 1027165813@qq.com +86 759 2620060

## Abstract

The effect of storage time on green coffee volatile organic compounds (VOCs) was studied by their separation *via* head space solid-phase microextraction and identification *via* gas chromatography-ion mobility spectrometry. In total, 38 kinds of VOCs, mainly composed of alcohols, aldehydes, esters and ketones, were identified. The fingerprint showed that the VOCs produced by green coffee in different years had obvious differences, especially, acrolein, 3-methylbutyl acetate, butanoic acid, heptan-3-ol, and so on, that could be used to predict the storage time. In addition, with the increase of storage time, the contents of butanal, ethanol, dimethyl sulfide, propanal, butan-2-one had no obvious change, and could be considered as typical aroma characteristics of green coffee or special aroma components for variety identification. Meanwhile, principal component analysis (PCA) and “nearest neighbor” fingerprint analysis could also effectively distinguish green coffee with different storage times. Comprehensive analysis showed that GC-IMS technology could provide strong and favorable support for coffee storage.

## Abstract 2

The effect of storage time on green coffee VOCs was studied by their separation *via* HS-SPME and identification *via* GC-MS.

## Graphical abstract

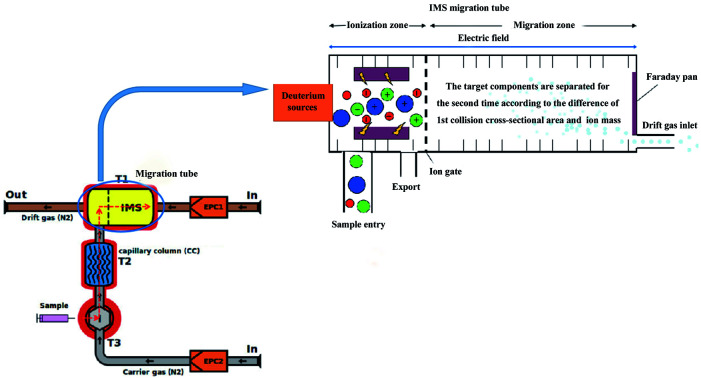

## 1 Introduction

Coffee is one of the three major beverages in the world, and the global coffee harvest area is 10.8401 million hectares in 2018. With the increasing demand for coffee in the world, the coffee production and trade in China have also made great progress. In 2018, the coffee planting area in china was 122 700 hectares, with a total output of 137 900 tons, which is more than five times higher than that in 2006, and the planting area and output of Yunnan Province accounts for more 98% of the whole country, and the coffee has become one of the eight plateau characteristic industries in Yunnan Province.^1,2^ Coffee not only contains caffeine, tannins, and other components beneficial to the body in terms of diuretic, central nervous system stimulation, and respiratory system, but its VOCs also have many health benefits. Pachimsawat *et al.*^3^ showed that VOCs could reduce salivary α-amylase (sAA) and cortisol (sCort) levels of patients during dental surgery (exploration and scraping), which proved that VOCs of coffee have a sedative effect.

The flavor of coffee is regarded as one of the important indexes to evaluate the quality and acceptability of coffee.^4^ The aroma of coffee is complex and volatile, composed of aldehydes, ketones, furanone, furan, sulfur-containing compounds, and pyrazine, and so on.^5^ For example, VOCs in Arabica coffee were dominated by furan and pyrazine derivatives.^6^ The aroma of coffee is not only related to the variety but also to the pretreatment process (dry or wet process),^7–9^ roasting method^10,11^ and other factors. Yu Fei *et al.*^12^ showed that three roasting methods, fast, medium and slow roasting, had an impact on the species and contents of VOCs. Juerg *et al.*^13^ showed that high temperature and roasting time had a significant effect on VOCs in coffee, with high temperature inducing 2,3-pentanedione, dimethyl trisulfide and other VOCs decreased significantly and 2-furfurylthiol, pyridine, *N*-methylpyrrole and other VOCs increased significantly. Li Na *et al.*^14^ showed that mild, moderate and deep roasting had significant effects on the VOCs of Robusta coffee. The overall aroma of mild roasted coffee beans was thin and the overall aroma of deep roasted was strong and burnt bitter *etc.* Ralph *et al.*^15^ found that ferulic acid decarboxylated, hydrolyzed and oxidized during coffee roasting to produce guaiacol and other phenolic volatile compounds. Chen *et al.*^16^ studied the effects of cold extraction and hot extraction on the aroma of Arabica coffee in Yunnan, and identified 111 kinds and 108 kinds of VOCs from the extracted coffee leaves, among which the content of furfuryl acetate in cold extraction was the highest. Sun^17^ revealed that the aroma intensity of freshly brewed coffee liquid will decrease significantly, and 2-furfuryl mercaptan and some characteristic aroma will fade rapidly during the placing process. According to the premise research, it was found that the aroma of green coffee was getting weaker due to the influence of temperature and humidity during storage, and few studies in this area had been reported.

**Fig. 1:**
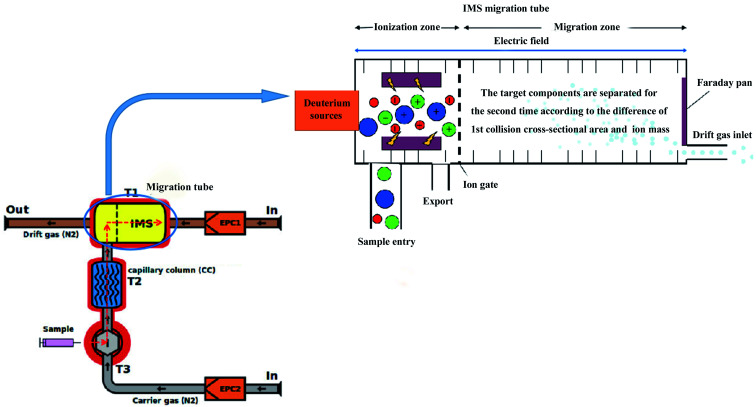
GC-IMS schematic diagram, it was provied by Shandong Hanon Instruments Co., Ltd.

The schematic diagram of gas chromatography-ion mobility spectrometry (GC-IMS) is shown in Fig. 1. It can be seen from Fig. 1 that the sample enters the instrument along with the carrier gas, which is first separated by the gas chromatography column and then enters the ion transfer tube according to the red dotted line. After being ionized in the ionization zone, the molecule to be measured migrates to Faraday disk for detection under the action of electric field and reverse drift gas, thus realizing secondary separation. Compared with the traditional gas chromatography-mass spectrometry (GC-MS) method, the GC-IMS method requires no pretreatment of the sample and the analysis is performed at a lower temperature, which can truly reflect the original state of the samples. It has been widely used in the fields of storage and processing of agricultural products,^18–20^ species identification and origin traceability.^21^ Based on the differences of VOCs fingerprinting by GC-IMS, the differentiation of coffee from different varieties^22,23^ and different origins^24^ had been successfully achieved at home and abroad, and it provided a certain reference and theoretical basis for variety identification, origin traceability and quality control of green coffee. In this study, GC-IMS was used to separate and identify the VOCs of green coffee from Arabica in Yunnan in different years, The VOCs were combined with thermogram analysis to reveal the changes of volatile aroma during the storage process of raw coffee, which could provide basic data to guide coffee storage, production and trade.

## 2 Materials and methods

### 2.1 Materials

Green coffee was provided by Yunnan International Coffee Trading Center and made by wet processing technology, and originated from the variety of Catimor in 2015, 2016, 2018, 2019 and 2020, which belongs to the Arabica coffee family. In addition, the green coffee in 2017 was not in stock, so no samples of other species of green coffee in 2017 were collected. The state of the green coffee was in grainy form for the measurement process. The experimental consistency and uniformity were better.

### 2.2 Conditions of head space gas chromatography ion mobility spectrometry

Head space gas chromatography ion mobility spectrometry (FlavourSpec 1H1-00206, G.A.S. Gesellschaft für analytische Sensorsysteme mbH) was described by Yijun *et al.*^19^ With some modification about the parameters. The green coffee samples (1.0 g each) was transferred to a 20 mL head vial and heated in an incubator at an incubator at an oscillation rate of 500 rpm, 60 °C for 20 min. Then, the samples were injected into a quartz capillary column (MXT-WAX, 30 m × 1 μm × 0.53 mm) by nitrogen at a programmed flow as follows: 2 mL min^−1^ for 2 min, 10 mL min^−1^ for 8 min, 100 mL min^−1^ for 30 min, and the syringe temperature was 85 °C and injection volume was 200 μL by a headspace automatic sampler (CTC CombiPAL, CTC Analytics AG). The compound were driven to the ionization chamber to be ionized in a positive ion mode by a 3H ionization source, and each spectrum was scanned 12 times on average. Then, the positive ions generated were separated in the drift tube for a second time, the length of the drift tube was 98 mm, the drift tube was operated at a constant temperature of 45 °C, and a voltage of 500 v cm^−1^. The drift gas (nitrogen) was set to 150 mL min^−1^. All analyses were performed in triplicate.

### 2.3 Statistical analysis

The instrumental analysis software includes LAV(Laboratory Analytical Viewer), three plug-ins and GC × IMS Library Search, in which LAV is used to view the analysis spectrogram, Reporter plug-in directly compare the spectrogram differences between samples (2D top view and 3D spectrogram), Gallery Plot plug-in fingerprint analysis and visually and quantitatively compare the VOCs differences between different green coffee samples, Dynamic PCA plug-in dynamic principal component analysis and quickly determine the types of unknown samples, and the NIST database and IMS database built in GC × IMS Library Search application software can conduct two-dimensional qualitative analysis of VOCs.

## 3 Results and discussion

### 3.1 Comparative analysis of GC-IMS topographic plots of VOCs produced from green coffee in different years

**Fig. 2:**
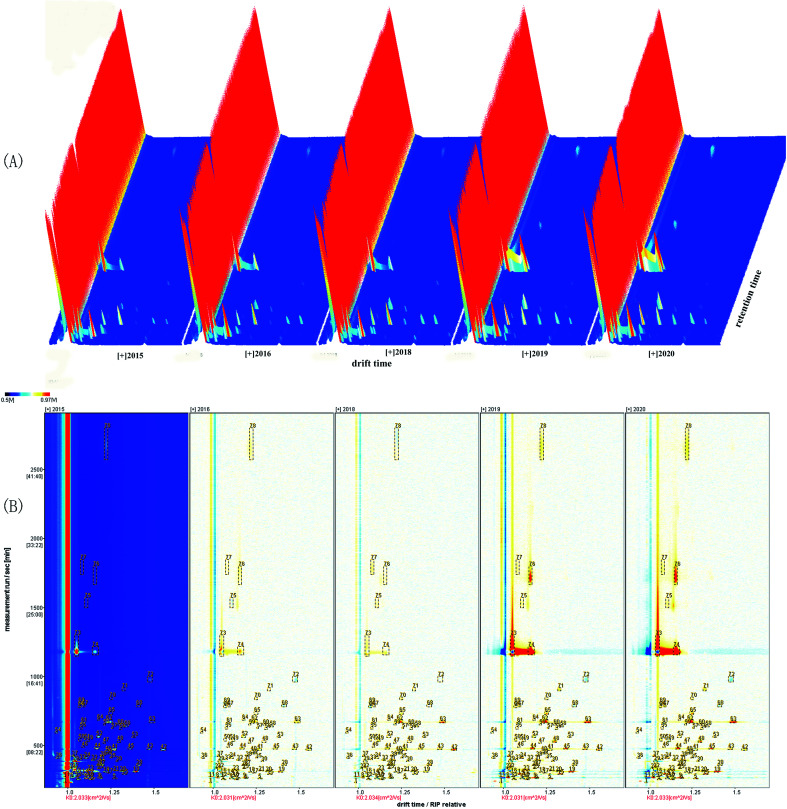
3D topographical visualization view of volatile organic compounds in green coffee of different years (A), and 3D topographical (top view) of volatile organic compounds in green coffee of different years (B).

The tree-dimensional pattern of the gas chromatographic ion mobility spectrogram of VOCs produced from green coffee in different years is shown in Fig. 2(A) and the top view is shown in Fig. 2(B). The relative retention index of each VOCs in the GC-IMS spectrum is shown in Table 1. The background of Fig. 2(A) is blue, the red vertical line at 1.0 on the abscissa is the reaction ion peak, and each dot on the right side represents a VOC. The color is indicative of the concentration of the VOCs, while white indicates a lower concentration and red indicates a higher concentration, and a darker color indicates a higher concentration. The difference comparison model was used to compare the differences in green coffee samples, as shown in Fig. 2(B). The figure was according to the spectrograms of green coffee in 2015. If the concentration of VOCs in the figure is the same, the colors cancel each other out to be white. The blue area in the reference sample indicates that the concentration of VOC is lower than that of the reference sample, and the deeper the blue, the lower the concentration. The red area in the reference sample indicates that the concentration of the substance is higher than that of the reference sample, and the deeper the red color, the higher the concentration.

**Table 1:** GC-IMS integration parameters of volatile organic compounds^d^

No	Compound	CAS#	Formula	MW	RI^a^	Rt [sec]^b^	Dt [a.u.]^c^	Identification approach	Classification
1	Dimethyl sulfide	C75183	C2H6S	62.1	789	233.901	0.98681	RI, DT	Thioethers
2	Propanal M	C123386	C3H6O	58.1	823.4	256.108	1.06252	RI, DT	Aldehydes
3	Propanal D	C123386	C3H6O	58.1	822.9	255.789	1.14011	RI, DT	Aldehydes
4	Butanal M	C123728	C4H8O	72.1	836	264.262	1.11086	RI, DT	Aldehydes
5	Butanal D	C123728	C4H8O	72.1	833	262.305	1.2804	RI, DT	Aldehydes
6	1	Unidentified	—	0	835	263.6	1.19543	—	—
7	1-Methylethyl acetate	C108214	C5H10O2	102.1	842.6	268.491	1.15266	RI, DT	Esters
8	Methyl acetate M	C79209	C3H6O2	74.1	848.2	272.09	1.0306	RI, DT	Esters
9	Methyl acetate D	C79209	C3H6O2	74.1	844.5	269.742	1.1871	RI, DT	Esters
10	2	Unidentified	—	0	856.3	277.31	1.12705	—	—
11	Acrolein M	C107028	C3H4O	56.1	858	278.426	0.98492	RI, DT	Aldehydes
12	Acrolein D	C107028	C3H4O	56.1	856.5	277.469	1.06098	RI, DT	Aldehydes
13	3	Unidentified	—	0	877.3	290.86	1.11937	—	—
14	Ethyl acetate M	C141786	C4H8O2	88.1	883.4	294.845	1.0921	RI, DT	Esters
15	Ethyl acetate D	C141786	C4H8O2	88.1	883.2	294.669	1.33346	RI, DT	Esters
16	Butan-2-one M	C78933	C4H8O	72.1	900.3	305.759	1.06112	RI, DT	Ketones
17	Butan-2-one D	C78933	C4H8O	72.1	898.3	304.411	1.24153	RI, DT	Ketones
18	Isopropanol	C67630	C3H8O	60.1	908.5	311.055	1.21861	RI, DT	Alcohols
19	3-Methylbutanal	C590863	C5H10O	86.1	911.9	313.225	1.39859	RI, DT	Aldehydes
20	*tert*-butanol	C75650	C4H10O	74.1	915.5	315.56	1.32225	RI, DT	Alcohols
21	4	Unidentified	—	0	919.8	318.302	1.27969	—	—
22	Ethanol M	C64175	C2H6O	46.1	925.2	321.801	1.04364	RI, DT	Alcohols
23	Ethanol D	C64175	C2H6O	46.1	924	321.018	1.12992	RI, DT	Alcohols
24	5	Unidentified	—	0	948.4	336.781	1.02026	—	—
25	6	Unidentified	—	0	947.2	335.978	1.18486	—	—
26	7	Unidentified	—	0	981.8	358.328	1.18097	—	—
27	Pentanal M	C110623	C5H10O	86.1	986.1	362.484	1.19875	RI, DT	Aldehydes
28	Pentanal D	C110623	C5H10O	86.1	985.5	361.726	1.41986	RI, DT	Aldehydes
29	8	Unidentified	—	0	1006.5	388.647	1.04075	—	—
30	9	Unidentified	—	0	1006.8	388.952	1.28979	—	—
31	Ethyl 2-methylpropanoate	C97621	C6H12O2	116.2	1012.3	395.98	1.19587	RI, DT	Esters
32	Butan-2-ol	C78922	C4H10O	74.1	1013.1	397.049	1.15042	RI, DT	Alcohols
33	1-Penten-3-one	C1629589	C5H8O	84.1	1016.9	401.938	1.08074	RI, DT	Ketones
34	Propan-1-ol M	C71238	C3H8O	60.1	1025.7	413.243	1.10982	RI,DT	Alcohols
35	Propan-1-ol D	C71238	C3H8O	60.1	1025.2	412.48	1.25464	RI, DT	Alcohols
36	10	Unidentified	—	0	1026.4	414.072	0.94288	—	—
37	11	Unidentified	—	0	1035.5	425.771	1.03408	—	—
38	12	Unidentified	—	0	1035.3	425.465	1.20011	—	—
39	Butyl acetate	C123864	C6H12O2	116.2	1049.2	443.247	1.23877	RI, DT	Esters
40	Ethyl 2-methylbutyrate	C7452791	C7H14O2	130.2	1061.2	458.652	1.23727	RI, DT	Esters
41	Hexanal M	C66251	C6H12O	100.2	1071.3	472.612	1.27316	RI, DT	Aldehydes
42	Hexanal D	C66251	C6H12O	100.2	1071.8	473.568	1.56315	RI, DT	Aldehydes
43	13	Unidentified	—	0	1073.1	476.169	1.47648	—	—
44	Isobutanol M	C78831	C4H10O	74.1	1075.7	481.212	1.171	RI, DT	Alcohols
45	Isobutanol D	C78831	C4H10O	74.1	1075.2	480.257	1.36625	RI, DT	Alcohols
46	14	Unidentified	—	0	1081.8	493.319	1.09304	—	—
47	15	Unidentified	—	0	1097.1	523.333	1.21486	—	—
48	3-Methylbutyl acetate	C123922	C7H14O2	130.2	1099.9	528.845	1.30152	RI, DT	Esters
49	4-Methyl-3-penten-2-one	C141797	C6H10O	98.1	1104.1	537.112	1.1224	RI, DT	Ketones
50	16	Unidentified	—	0	1107.1	542.933	1.07423	—	—
51	(*E*)-2-pentenal	C1576870	C5H8O	84.1	1110.2	549.058	1.10448	RI, DT	Aldehydes
52	1-Butanol M	C71363	C4H10O	74.1	1119.4	567.211	1.18336	RI, DT	Alcohols
53	1-Butanol D	C71363	C4H10O	74.1	1118.9	566.256	1.3852	RI, DT	Alcohols
54	1-Penten-3-ol	C616251	C5H10O	86.1	1133.9	595.771	0.93615	RI, DT	Alcohols
55	17	Unidentified	—	0	1149.1	625.623	1.08241	—	—
56	18	Unidentified	—	0	1147.4	622.234	1.30772	—	—
57	2-Heptanone	C110430	C7H14O	114.2	1150	627.461	1.25901	RI, DT	Ketones
58	Heptanal	C111717	C7H14O	114.2	1157.1	640.936	1.34403	RI, DT	Aldehydes
59	Limonene M	C138863	C10H16	136.2	1165.1	654.411	1.21404	RI, DT	Alkenes
60	Limonene D	C138863	C10H16	136.2	1164.3	653.186	1.29988	RI, DT	Alkenes
61	19	Unidentified	—	0	1171.1	664.677	1.09192	—	—
62	3-Methyl-1-butanol M	C123513	C5H12O	88.1	1176.7	674.01	1.24541	RI, DT	Alcohols
63	3-Methyl-1-butanol D	C123513	C5H12O	88.1	1177	674.512	1.49281	RI, DT	Alcohols
64	20	Unidentified	—	0	1185.4	688.712	1.17888	—	—
65	Pentanol	C71410	C5H12O	88.1	1212.4	734.303	1.25276	RI, DT	Alcohols
66	Dihydro-2-methyl-3(2H)-furanone	C3188009	C5H8O2	100.1	1240.6	781.732	1.07297	RI, DT	Ketones
67	21	Unidentified	—	0	1243.4	787.328	1.09304	—	—
68	Hexyl acetate	C142927	C8H16O2	144.2	1244.3	789.165	1.41189	RI, DT	Esters
69	22	Unidentified	—	0	1252.5	806.576	1.07214	—	—
70	23	Unidentified	—	0	1268.8	841.379	1.25748	—	—
71	Heptan-3-ol	C589822	C7H16O	116.2	1300.6	908.979	1.32987	RI, DT	Alcohols
72	24	Unidentified	—	0	1333	977.867	1.48121	—	—
73	Acetic acid M	C64197	C2H4O2	60.1	1434.5	1193.909	1.04924	RI, DT	Acids
74	Acetic acid D	C64197	C2H4O2	60.1	1427.9	1179.901	1.16118	RI, DT	Acids
75	25	Unidentified	—	0	1595.9	1537.169	1.11253	—	—
76	Butanoic acid	C107926	C4H8O2	88.1	1682.6	1721.763	1.158	RI, DT	Acids
77	3-(Methylthio)-1-propanol	C505102	C4H10OS	106.2	1709.7	1779.34	1.08316	RI, DT	Alcohols
78	4-Methoxyacetophenon	C100061	C9H10O2	150.2	2127.9	2668.902	1.22641	RI, DT	Ketones

a Represents the retention index calculated using n-ketones C4–C9 as external standard on FS-SE-54-CB-1 column.

b Represents the retention time in the capillary GC column.

c Represents the drift time in the drift tube.

d D represents a dimer, and M represents a monomer.

It could be seen from the Fig. 2(A) and (B) that the retention time of VOCs released from green coffee was 100–2000s, and the signal peaks with strong differences were concentrated in 1000–2000s. There were differences in VOCs released by green coffee in different years, and the shorter the year, the more peaks and the greater the peak intensity, and most VOCs gradually disappear during storage. The fresher the green coffee, the higher the amount of these compounds detected, and the most VOCs emitted by green coffee (new green coffee) in 2020.

**Fig. 3:**
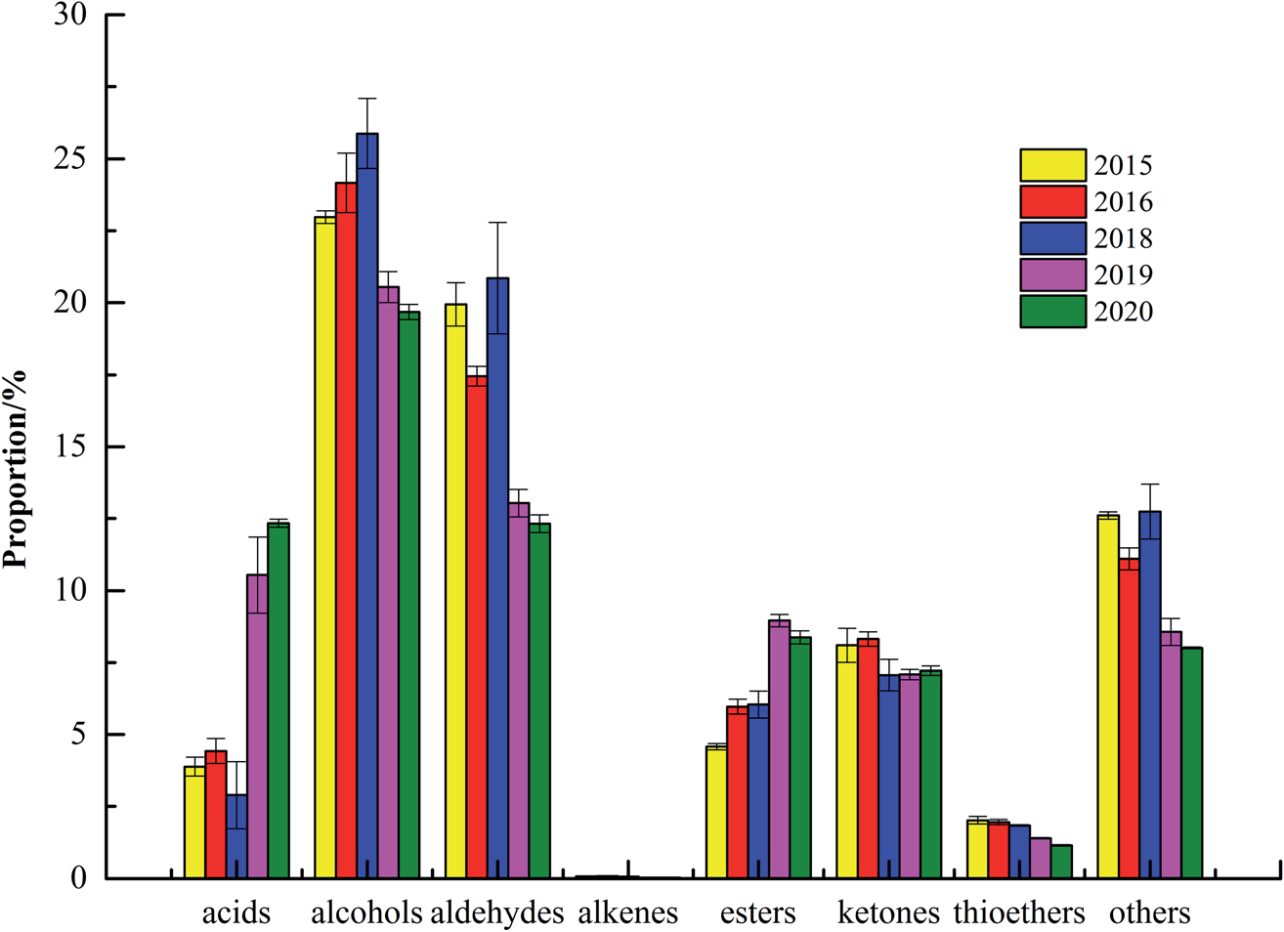
The percentage of the content of volatile organic compounds in green coffee in different years.

Table 1 and Fig. 3 showed that 78 kinds of VOCs were detected, including 15 kinds of monomolecular compounds, 25 kinds of unidentified components, and 38 kinds of VOCs were qualitatively detected through built-in NIST database and IMS database. The 38 kinds of VOCs of green coffee were mainly alcohols, aldehydes, esters and ketones, including 12 kinds of alcohols, 8 aldehydes, 8 esters, 6 ketones, 2 acids, 1 alkene and 1 thioether. Due to the differences in varieties, sampling amount and time, there were the differences in the types and numbers compared to the reported by Du Ping *et al.*, but more than 80% of the VOCs were similar.^24^ Meanwhile, the VOCs in green coffee were changed after roasting, which were also the reason for the difference.^6^ The results of significance analysis showed that the contents of acids and esters in fresh green coffee (in 2020) changed significantly from 2019 to 2015, alcohols, aldehydes and alkenes changed significantly from 2018 to 2015, and ketones changed significantly from 2016 to 2015.

### 3.2 Comparative analysis of GC-IMS fingerprint spectra of VOCs produced from green coffee in different years

**Fig. 4:**
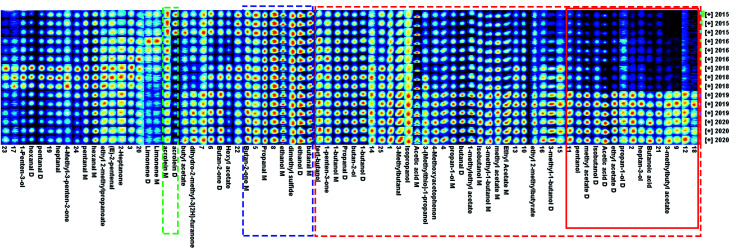
Fingerprint spectra of volatile organic compounds in green coffee of different years.

The fingerprint information of VOCs produced by green coffee in different years was shown in Fig. 4. Each row in Fig. 4 represented all signal peaks selected in one sample, and each column represented the signal peaks of the same VOCs in different samples. Color represented the concentration of compounds, where white indicated lower concentration and red indicates higher concentration, and a darker color indicates higher concentration.

As could be seen from Fig. 4, methyl acetate, ethyl acetate, ethyl 2-methylbutyrate, 3-methylbutyl acetate, 1-methylethyl acetate, isopropanol, propan-1-ol, butan-2-ol, 1-butanol, isobutanol, *tert*-butanol, pentanol, heptan-3-ol, 3-(methylthio)-1-propanol, 3-methyl-1-butanol, propanal, 3-methylbutanal, butanal, acetic acid, butanoic acid, 1-penten-3-one, 4-methoxyacetophenon and other substances were higher in green coffee with shorter years, and lower with longer years. The 3-methylbutyl acetate, Butanoic acid, heptan-3-ol and other substances in the red solid frame decreased rapidly from 2018. The variation law of acrolein in the green dotted box was just the opposite, and the longer the year, the higher the content. Therefore, it could be inferred that Acrolein could be used as the basis for characterizing the age of green coffee. Acrolein had the risk of being converted into acrylamide which was harmful to human body,^25^ so it had special significance for guiding the storage and consumption of green coffee. The contents of butanal, ethanol, dimethyls sulfide, propanal and butan-2-one in the blue dotted box did not change obviously with the time of year. Therefore, it could be considered that the compounds in the blue dotted frame were the characteristic compounds that distinguish Arabica coffee from other varieties and producing areas. Under the action of high temperature, the VOCs of roasted coffee were quite different from those of green coffee, especially furans,^26^ and related reports show that pretreatment process and roasting process conditions also had great differences in coffee aroma,^27,28^ so it was more meaningful to identify varieties and producing areas based on the aroma characteristics of green coffee.

### 3.3 Trend analysis of VOCs produced from green coffee in different years

**Fig. 5:**
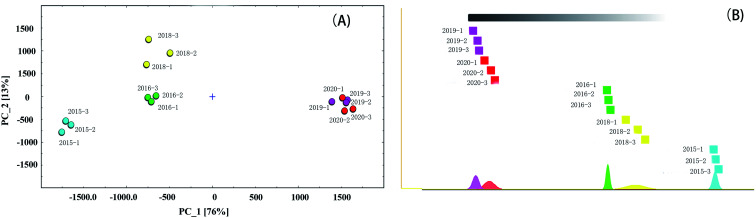
PCA plot of VOCs produced from green coffee in different years (A) and The “nearest neighbor” fingerprint plot of VOCs produced from green coffee in different years (B).

Principal component analysis (PCA) is a statistical method of dimension reduction. It is a statistical method of transforming the original random vector related to components into new random vectors unrelated to components by means of orthogonal transformation, and the new random variables reflect the information of the original variables as much as possible. This method has been widely used in classification analysis and quality evaluation of agricultural products.^29–32^ In order to highlight the differences of VOCs in different green coffee samples, PCA was used for cluster analysis, as shown in Fig. 5(A). It could be seen from Fig. 5(A) that the first and second main component variance contribution rates were 76% and 13%, respectively, and these components showed the similarity among the different green coffee samples.

It could be seen from Figure that the green coffee in 2015, 2016 and 2018 could be well distinguished in the distribution map, while the VOCs of green coffee in 2019 and 2020 were similar and could not be well distinguished. When the value of PC_1 was less than −1500, it could be judged as green coffee in 2015. When the value of PC_1 was greater than −1000 and less than 0, and the value of PC_2 was greater than −250 and less than 250, it could be judged as green coffee in 2016. When the value of PC_1 was greater than −1000 and less than 0, and the value of PC_2 was greater than 500, it could be judged as green coffee in 2018. When the value of PC_1 was greater than 1000, it could be judged as green coffee in 2019 or 2020 (fresh green coffee).

The “nearest neighbor” fingerprint analysis based on Euclidean distance algorithm is to compare samples quickly according to the intensity of compounds in the selected evaluation area. The algorithm calculates the Euclidean distance between every two samples. In this way, you can find the “nearest neighbor” by retrieving the minimum distance.^33^ Firstly, two measured values of the farthest Euclidean distance (minimum similarity) of the two measured values are determined. A box representing the measured value was drawed and placed on the leftmost side. Then, its “nearest neighbor” is placed on the right side, and so on, until all measured values are displayed. The bottom area of the graph represents the normal distribution of each sample (color). The method to interpret the map is not to find the farthest, but to observe the relatively close group measurement results compared with the farther group. The “nearest neighbor” map of green coffee in different years was shown in Fig. 5(B). It could be seen from Fig. 5(B)that there were certain differences in VOCs released by green coffee in different years, among which the green coffee in 2015 were obviously different from other years, the green coffee in 2018 and 2016 were some different but not very big, and the green coffee in 2019 and 2020 were very similar. It could be inferred that the VOCs released from green coffee begun to change significantly after the second year of storage.

## 4 Conclusion

In this study, 38 kinds of VOCs, composed of alcohols, aldehydes, esters and ketones, were identified by GC-IMS in green coffee from different years. The VOCs released by green coffee were affected by different storage time. From the third year, 3-methylbutyl acetate, Butanoic acid and heptan-3-ol in most green coffee decreased rapidly. Meanwhile, the longer the year, the higher the content of acrolein in VOCs, so the storage life of green coffee could be inferred by detecting the changes of these compounds. In addition, acrolein had the risk of being converted into acrylamide which was harmful to human body, so it was suggested that the storage period of green coffee should be less 2 years. The contents of butanal, ethanol, dimethyls sulfide, propanal and butan-2-one did not change obviously with the years, they could be used as typical VOCs of green coffee or as characteristic aroma components for variety identification. PCA and nearest neighbor fingerprint analysis could effectively distinguish green coffee in different years and provided basic data support for green coffee storage.

## 5 Author contributions

Chen Min: Writing - original draft, Conceptualization, Investigation. Mai Biyi: Data curation, Formal analysis, Methodology. Lu Jianneng: Data curation, Formal analysis. Li Yimin:Data curation. Liu Yijun: Writing - review & editing, Validation, Methodology, Funding acquisition. Cheng Long: Validation, Methodology, Writing - review& editing.

## Conflicts of interest

The authors declare no conflict of interest. The founding sponsors had no role in the study's design, in the collection, analyses, or interpretation of data, in the writing of the manuscript, and in the decision to publish the results.

## Supplementary Material
